# Twins' Health and Adult Socioeconomic Status

**DOI:** 10.1371/journal.pmed.0020235

**Published:** 2005-07-26

Twins' research is a favorite tool of the human geneticist, but it has a controversial history. Nazi doctor Josef Mengele, infamous for his work at Auschwitz, was fascinated by twins. He sought them out at the extermination camp and used them in violent experiments. Later, British psychologist Cyril Burt worked on the heredity of intelligence, producing findings that some suspected were “too good”—and which later were shown to be based on fraudulent data involving invented twins.

In this month's *PLoS Medicine*, Nancy Krieger and colleagues examine the health of female twins from a very different perspective. To understand the impact of lifetime socioeconomic position on health, they studied twins who were raised together but who had different socioeconomic position after adolescence. Many studies have compared the health of twins raised separately since birth or early childhood, but few have investigated the adult health of twins raised together but who had different post-adolescent socioeconomic position. Such a study could clarify the impact of adult experiences on adult health in a population matched on early life experiences, the authors say.

Krieger's team employed data from a cohort of 434 twins who lived in the San Francisco Bay area in 1978–1979; the average age at recruitment was 41 years old. The cohort was given a health and sociodemographic questionnaire. Data on anthropometric and biological characteristics were obtained by physical examination and laboratory analyses; the process was repeated at a second examination around 1989–1990. At the second phase of the study, 72 women (8.3%) did not return, of whom 36 had died, which left 352 twin pairs (58% monozygotic, 42% dizygotic), representing 81.1% of the original cohort.

The sociodemographic and health characteristics of the full cohort (352 pairs) and the cohort analyzed (those pairs where it was known both that they had lived together until at least age 14 and their joint socioeconomic was available—i.e., 308 pairs) were quite similar: 40% grew up in working-class households and 80% in households where the father had fewer than four years of college education.

At the second examination, 32% of the twin pairs in the analytic cohort had a difference in their adult household occupational class, and 20% had different levels of college education. The team found that health outcomes among monozygotic adult female twins who lived together through childhood varied by their subsequent socioeconomic position. Twins with differing occupational class differed in health status compared with twins with similar occupational class. The working-class twin had significantly higher systolic blood pressure, diastolic blood pressure, and LDL cholesterol than her professional, non-working-class twin. Twins discordant on educational level, however, had similar health status, likely reflecting the fact that current occupational class is a better measure for investigating the impact of lifetime socioeconomic position than is education (which is completed usually in early adulthood). Dizgyotic twins with differing adult socioeconomic position, either occupational class or educational level, did not notably differ in their adult health status.

These novel findings lend support to the hypothesis that health is shaped not only by early life experiences but also by cumulative experiences across the lifecourse, the authors say.

As with many twin studies, the numbers of individuals studied is relatively limited. The lack of some biological and personal data such as detailed occupational class position over time, lack of data on income, poverty, wealth, debt, gestational age, birthweight, and birth order also limits the conclusions that can be drawn. These limitations are countered, however, by the tight matching of the twins on early life and childhood exposures, as well as by the matching for genetic endowment among the monozygotic twins. Together, the findings have important implications for health policy, since they suggest that adult socioeconomic position does have an impact on adult health above and beyond early life exposures, and they also add to our understanding of how societal conditions shape population patterns of health, disease, and well-being.[Fig pmed-0020235-g001]

**Figure pmed-0020235-g001:**
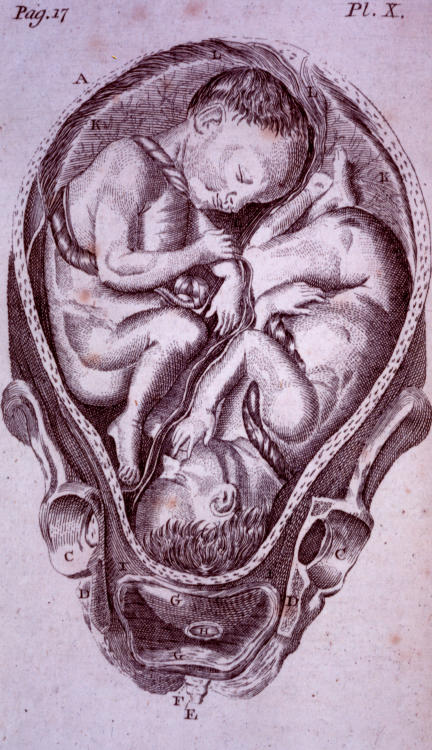
Twins in the womb (from the first US textbook on midwifery, *An Abridgement of the Practice of Midwifery*, by W. Smellie, 1786)

